# Dynamic Light Scattering on Bioconjugated Laser Generated Gold Nanoparticles

**DOI:** 10.1371/journal.pone.0089048

**Published:** 2014-03-13

**Authors:** Massimo Zimbone, Pietro Baeri, Lucia Calcagno, Paolo Musumeci, Annalinda Contino, Maria Luisa Barcellona, Gabriele Bonaventura

**Affiliations:** 1 Dipartimento di Fisica ed Astronomia, University of Catania, Catania, Italy; 2 Dipartimento di Scienze Chimiche, University of Catania, Catania, Italy; 3 Dipartimento di Scienze del Farmaco, University of Catania, Catania, Italy; University of Catania, Italy

## Abstract

Gold nanoparticles (AuNPs) conjugated to DNA are widely used for biomedical targeting and sensing applications. DNA functionalization is easily reached on laser generated gold nanoparticles because of their unique surface chemistry, not reproducible by other methods. In this context, we present an extensive investigation concerning the attachment of DNA to the surface of laser generated nanoparticles using Dynamic Light Scattering and UV-Vis spectroscopy. The DNA conjugation is highlighted by the increase of the hydrodynamic radius and by the UV-Vis spectra behavior. Our investigation indicates that Dynamic Light Scattering is a suitable analytical tool to evidence, directly and qualitatively, the binding between a DNA molecule and a gold nanoparticle, therefore it is ideal to monitor changes in the conjugation process when experimental conditions are varied.

## Introduction

Gold nanoparticles (AuNPs) have attracted large attention in the last decade due to their unique physical and chemical properties. In fact, AuNPs, besides their unique optical characteristics, chemical stability, low toxicity and biocompatibility [1]–[2], they also show the possibility to easily modify their surface by functionalization or conjugation with biomolecules [3]. Thus, they have been widely used either as analytical tools, either for biological and medical application, especially when they are conjugated with biomolecules. Target specificity, noninvasiveness, high spatial resolution, reactivity toward living cells and real-time imaging are some of the important requirements for biomedical technologies, where accurate and real-time imaging of biological targets is essential not only to understand the fundamental biological processes being between, but also to successfully diagnose various diseases. For example, DNA conjugated AuNPs play an important role in biosensing and in nanobiotechnology, and they find many applications in molecular diagnostic [4]–[7], nanofabrication [8], molecular nanoelectronic [9], cell imaging and gene regulation [10]–[13]. Concerning this last possible use, nanoparticles based probes play an important role not only in enhancing imaging sensitivity and resolution but also in possessing “molecular imaging” capability [14]–[15]. One of the advantages of nanoparticle probes is their flexibility when conjugated with DNA, peptides, and antibodies for monitoring specific molecular events such as gene expression and other biological processes as cell metastasis.

A crucial step in developing even more efficient biosensors is to establish simple protocols for the fast and reproducible synthesis of stable DNA/AuNPs conjugates. The gold conjugation is mediated by a thiol molecule which is previously attached to DNA oligonucleotides in the 3′ or 5′ end of the strand. Several studies have been reported in the past decade concerning the successful conjugation of DNA/AuNPs where the gold nanoparticles are synthesized by chemical methods [16]–[18], i.e. by reducing Au(III) compounds with the addition of a reduction agent, usually citrate, whose excess also works as a stabilizing agent either adsorbed or chemically bound to the surface of AuNPs. These processes give AuNPs covered by an ionic layer that prevents the particles aggregation.

A widely accepted protocol to immobilize DNA on AuNPs surface, was initially developed by Storhoff and co-workers [19]. In their approach, a two days incubation process was needed to link alkanethiol-terminated oligonucleotides and citrate-capped AuNPs. The method includes the addition of NaCl (0.1 M) to the reaction mixture to shield the negative charge of both DNA and nanoparticles and thus to minimize the repulsion between them, by obtaining DNA conjugated nanoparticles. However this process results in low DNA loading, too long reaction times (40 hrs) and the conjugation of DNA to nanoparticles is always in competition with the nanoparticle aggregation process induced by salt adding.

The same authors improved the method [20]: to avoid a salt-aided irreversible aggregation, the NaCl solution was gradually increased, from 0 to 0.3 M by several successive additions, and after each addition the solution was incubated for 16 hrs (“salt aging” procedure). The process could take several days allowing the chemical adsorption of oligonucleotides to the nanoparticle surface, estimated as 0.1 DNA-strands/nm^2^. In 2006, S. J. Hurst and co-workers [21] obtained the same surface coverage by optimizing all the experimental parameters and by using surfactant molecules, even if the stepwise addition of NaCl is still required but, at least, the all procedure takes only one day.

An alternative method to obtain bioconjugated gold nanoparticles in one step process is the pulsed laser ablation in liquid (PLAL) [22]–[25]. This technique allows generating ultrapure, electrostatically stabilized and highly efficient AuNPs with a “naked” surface. According to Sylvestre et al. [26] the particles produced by PLAL are partially oxidized, thus resulting more reactive and acting as electron acceptors, and therefore easily coordinated by molecules bearing electron donor moieties as thiol.

DNA conjugation of laser generated AuNPs has been extensively studied by Petersen et al. [27]. These authors obtained conjugation both “in situ”, i.e. by producing AuNPs in a solution containing thiolated DNA oligos, and “ex situ”, i.e. by adding DNA after the laser generated AuNPs process. The “in situ” conjugation leads to about four time higher conjugation efficiency than the “ex-situ” one. However, in the “ex-situ” conjugation, these authors do not use salt, but the DNA/AuNPs solution was incubated for 24 hrs. In this experiment a surface coverage of about 1.1 DNA-strands/nm^2^ was reached whereas the oligos/nanoparticle surface ratio in the solution exceeds 20 DNA-strands/nm^2^.

In some previous papers, the determination of DNA loaded on AuNPs was performed by dithiothreitol (DTT) displacement, followed by fluorescence measurements [28]–[29].

In the present work, we investigated the ex-situ conjugation of laser generated gold nanoparticles with DNA in presence of NaCl, by using Dynamic Light Scattering (DLS), UV-Vis spectroscopy. The influence of salt adding in the “aging” and “not aging” procedures is also investigated in details and both procedures are studied in presence of different DNA concentrations.

## Material Characterization and Experimental Methods

### 2.1 Gold Nanoparticles

Gold nanoparticles produced in water by PLAL are purchased by Particular GmbH (Germany). UV-Vis spectra were carried out by a Perkin-Elmer Lambda 35 spectrometer in the wavelength range 400–1100 nm.

The as-prepared samples were characterized by DLS (Dynamic Light Scattering) technique, which is sensitive and precise in detecting changes in the average particle dimensions.

The measurements were carried out by a homemade apparatus that comprises a quartz scattering cell, confocal collecting optics, a Hamamatzu photomultiplier mounted on a rotating arm, a BI-9100 AT hardware correlator (Brookhaven Instruments Corporation). The samples were lighted with a 660 nm diode laser whose power ranged between 15 and 150 mW. The intensity of the scattered light fluctuates over time due to the Brownian motion of the particles suspended in solution. The analysis of the scattered light fluctuations was performed by the intensity auto-correlation function (g_2_). This function was provided by the hardware correlator operating in single photon counting regime. For monodisperse non-interacting particles in Brownian motion, the g_2_ function is a decreasing exponential with a relaxation rate Γ equal to Γ = 1/τ with τ = decay time. Once the relaxation rate is obtained, it is possible to calculate either the translational diffusion coefficient (*D_t_*) and the hydrodynamic radius (*R_h_*) using the following expressions [30]–[31]:
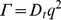
(1)where *q* is the scattering vector, defined as *q = (4πn/λ)sin(θ/2)*, being *n* the refraction index of the solvent, *λ* the light wavelength, *θ* the scattering angle,and
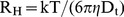
(2)where η is the liquid viscosity, k the Boltzmann constant and T the absolute temperature. We used a scattering angle θ = 90°. In polydisperse solutions, like in the case of the present manuscript, g2 shows several exponential decay components. The analysis of autocorrelation function g2 is then performed either with a cumulant or a multiexponential method [32]. The result is a z-average diffusion coefficient and hence, by eq. 2, a z-average hydrodynamic radius.

We have recently reported [33]–[34] that this technique is suitable to determine the size as well as the shape of gold nanoparticles and nanoaggregates.

### 2.2 DNA Functionalization

Single strained oligonucleotides (DNA) were purchased from Purimex GmbH (Germany) and the nucleotides sequence was: HS5′TGC ATG CAT GCA TGC ATG CAT GCA TGC ATG CAT GCA TGC ATG CAT GCA TC (50 Mer). The molecular weight and the concentration were 1.5558 kDa and 1.0 mM, respectively.

Firstly, the DNA was diluted in pure Milli-Q, Millipore Ultrapure water, with resistivity of 25 MΩ×cm, at two intermediated concentrations of 100 μM and 10 μM. The concentration value was determined by UV-vis measurements by using the absorbance value at 260 nm for single stranded DNA. In that measurement, 1 OD of absorbance corresponds to 2.24 nmol/ml of oligonucleotides.

In order to obtain AuNPs functionalized with DNA, we added a DNA solution to 1 ml of AuNPs solution to obtain three different DNA concentrations: 1.8, 0.7 and 0.1 μM respectively, and from now on we will refer in terms of “high”, “medium” and “low” for these three concentrations.

In order to test the DNA concentration in these DNA/AuNPs solution, we added the same amount of DNA in 1 ml of pure water and measured the absorbance at 260 nm. In fact, although the DNA absorption is negligible above 350 nm and it does not affects the plasmonic resonance of AuNPs, the absorption of the AuNPs solutions, in the range between 200 and 300 nm, makes difficult the evaluation of DNA absorption peak.

Subsequently, the functionalization process was accomplished by adding NaCl to the DNA/AuNPs solution by two different procedures, “salt aging” and “no salt aging”, respectively.

In the “salt aging” procedure we added 20 μl of a 1.0 M NaCl solution to 1.0 ml of the DNA/AuNPs solution, obtaining a sodium chloride concentration of 20 mM. Salt addition was repeated every eight hours until a final NaCl concentration of 100 mM was reached. In the “no salt aging” procedure the NaCl concentration was increased in the same way, but without standing for eight hours.

## Results and Discussion

The Surface Plasmon Resonance (SPR) of a colloidal gold solution originates an extinction spectrum depending on size, shape and aggregation level of nanoparticles and it is widely used for their characterization [35]–[37]. The UV-Vis spectrum of the as-prepared gold nanoparticle solution, generally, shows a strong absorption band with a maximum at 522 nm, characteristic of the collective absorption of the free conduction band electrons of the nanoparticles [38]–[40]. If aggregation of nanoparticles occurs the spectrum modifies, in particular a second red-shifted band appears at 650 nm.

As unerlined in the introduction, the screening of nanoparticles negative charged, induced by salt adding, promotes their aggregation, event to be avoided, since it is in competition with the DNA conjugation [41]–[42]. In order to evaluate the effects of salt adding on as-prepared AuNPs, without DNA, we have investigated the properties of colloidal gold solutions obtained by laser ablation as a function of NaCl concentration by using UV-vis absorption spectroscopy and DLS technique.

**Figure 1:**
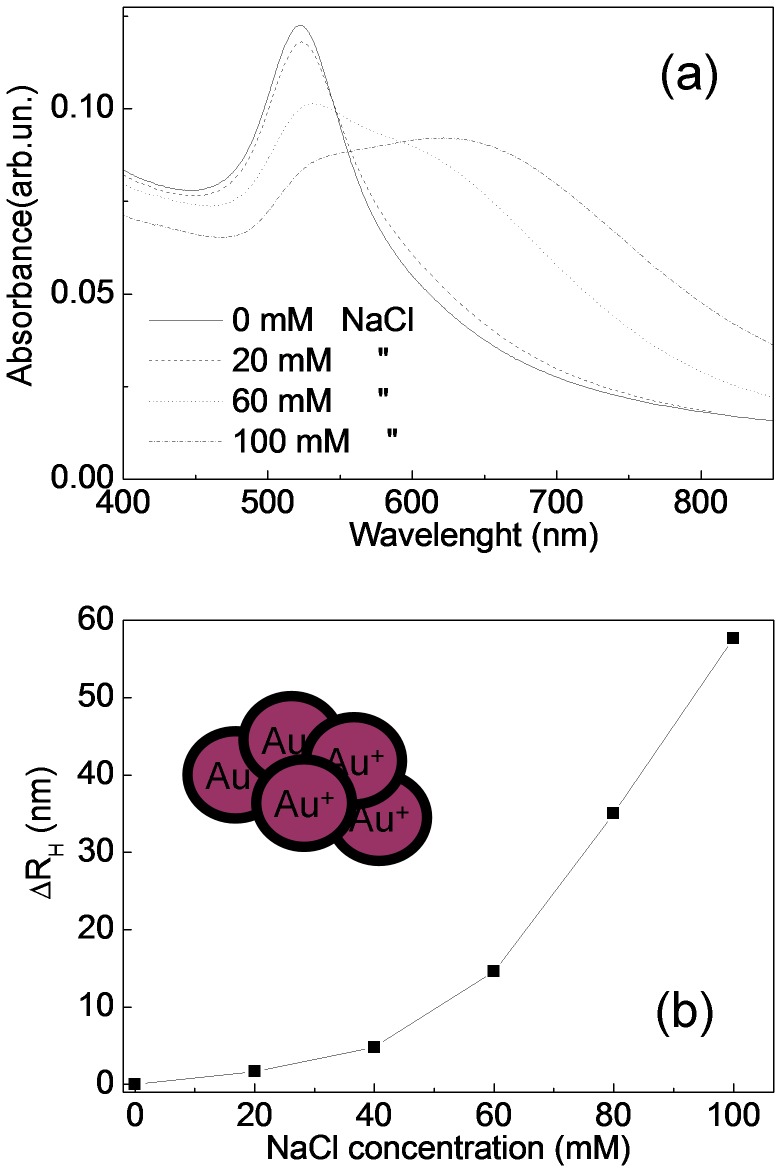
UV-Vis spectra of AuNPs aggregated with different NaCl concentrations (a). Hydrodynamic radius difference between aggregated and as-prepared nanoparticles measured with DLS as a function of NaCl concentration (b).

Fig. 1a reports the experimental extinction spectra in the wavelength range between 400 and 850 nm of as-prepared AuNPs colloid as well as of colloids obtained after salt solution addition at different concentrations. The extinction spectrum of as-prepared AuNPs solution shows a strong absorption band with a maximum at 522 nm. The addition of NaCl induces a significant change in the absorption spectra. In particular, increasing the salt concentration, the plasmon resonance peak at 522 nm decreases in intensity and a new absorbance band at longer wavelength increases, indicating the aggregation of the nanoparticles, due to their negative charges screening, which makes less efficient the repulsive forces among them.

We also characterized our samples by DLS technique. The autocorrelation functions of AuNPs (g_2_) significantly changes as the salt concentration increases: in particular the decay time increases evidencing the formation of larger nanoparticles. In Fig. 1b we report the differences (ΔR_H_) between the hydrodynamic radii of the aggregated samples and of the as-prepared AuNPs ones, as a function of salt concentration. The measured particle size difference increases with increasing the NaCl concentration, indicating the formation of nanoparticle clusters, in agreement with UV-Vis spectroscopy and with the results reported by Jans et al. [Jans et al. 2009] for gold nanoparticles prepared by Turkevich method [43].

Gold nanoparticles aggregation induced by salt addition can be inhibited by DNA loading on their surface. DNA loading is a slow process but it is irreversible and it gives to the nanoparticles a greater negative charge, increasing their stability and preventing aggregation also in the presence of high salt concentration. Moreover, AuNPs aggregation can occur before the attachment of DNA. To prevent nanoparticles aggregation, NaCl salt is added to the DNA/AuNPs solution with the “salt aging” process. The key point of this procedure is to wait for DNA to adhere to AuNPs, by allowing a charge increase on their surface and thus preventing the nanoparticle aggregation through a further repulsion between them [44]–[46].

We have investigated the DNA conjugation of gold nanoparticles at different DNA concentrations, i.e. “high”, “medium” and “low”, as indicated in the previous section. We have adopted both “salt aging” and “no salt aging” procedures.

**Figure 2:**
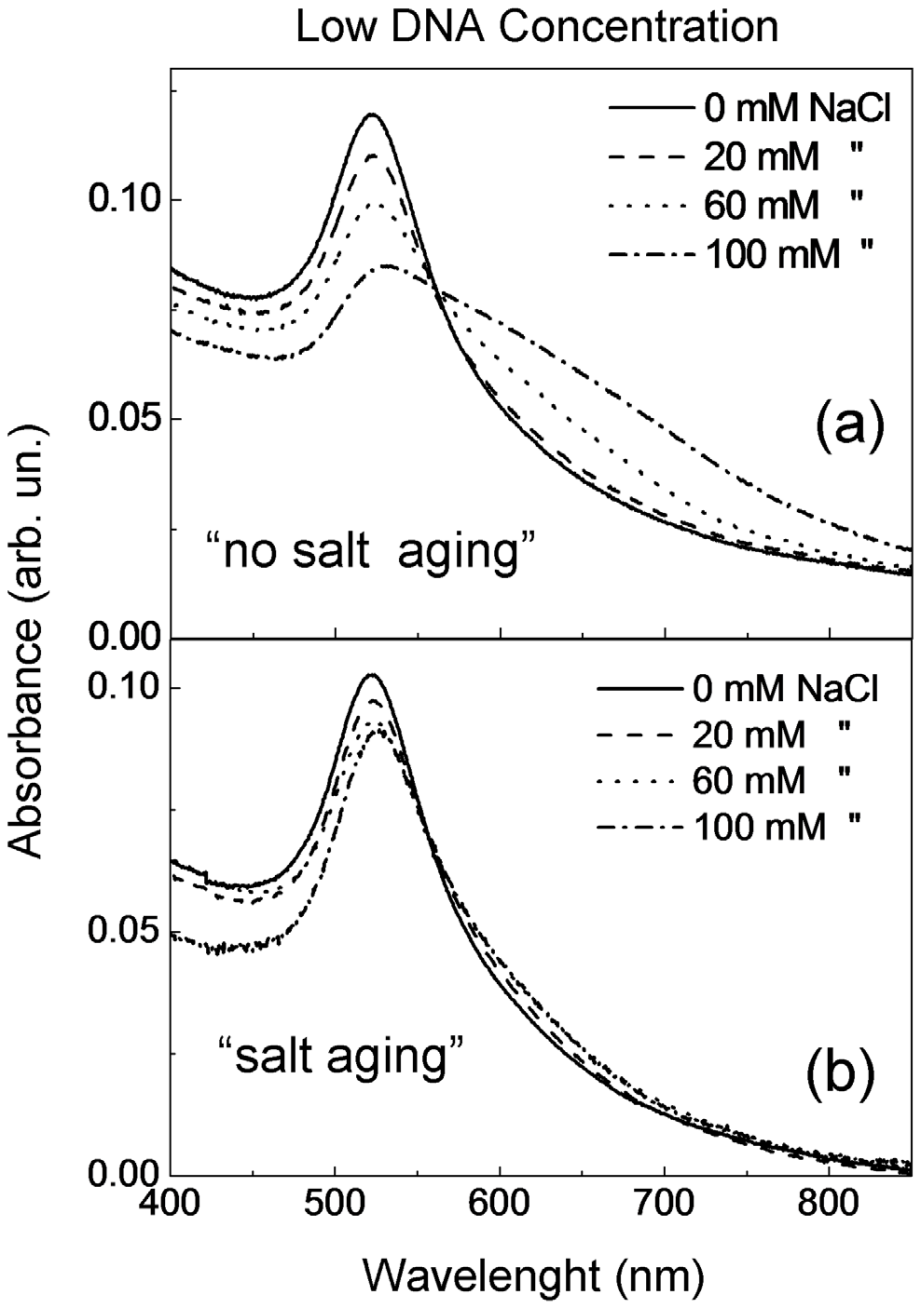
UV-vis spectra of AuNPs/DNA solution for different NaCl concentrations obtained with “no salt aging” (a) and “salt aging” (b) procedure. The DNA concentration was 0.1 μM.

The extinction spectra of AuNPs with “low” DNA, in presence of different NaCl concentrations are shown in Fig. 2. The results reported in Fig. 2a (“no salt aging”) show the appearance of a red-shifted band at NaCl concentrations higher than 20 mM, indicating the aggregation of the nanoparticles due to their negative charges salt screening that, finally, makes less efficient the repulsive forces among them. Instead the “salt aging” procedure (Fig. 2b) shows a very low change in the UV spectra up to highest NaCl concentration employed (100 mM), evidencing that the “salt aging” procedure prevents nanoparticle aggregation.

**Figure 3:**
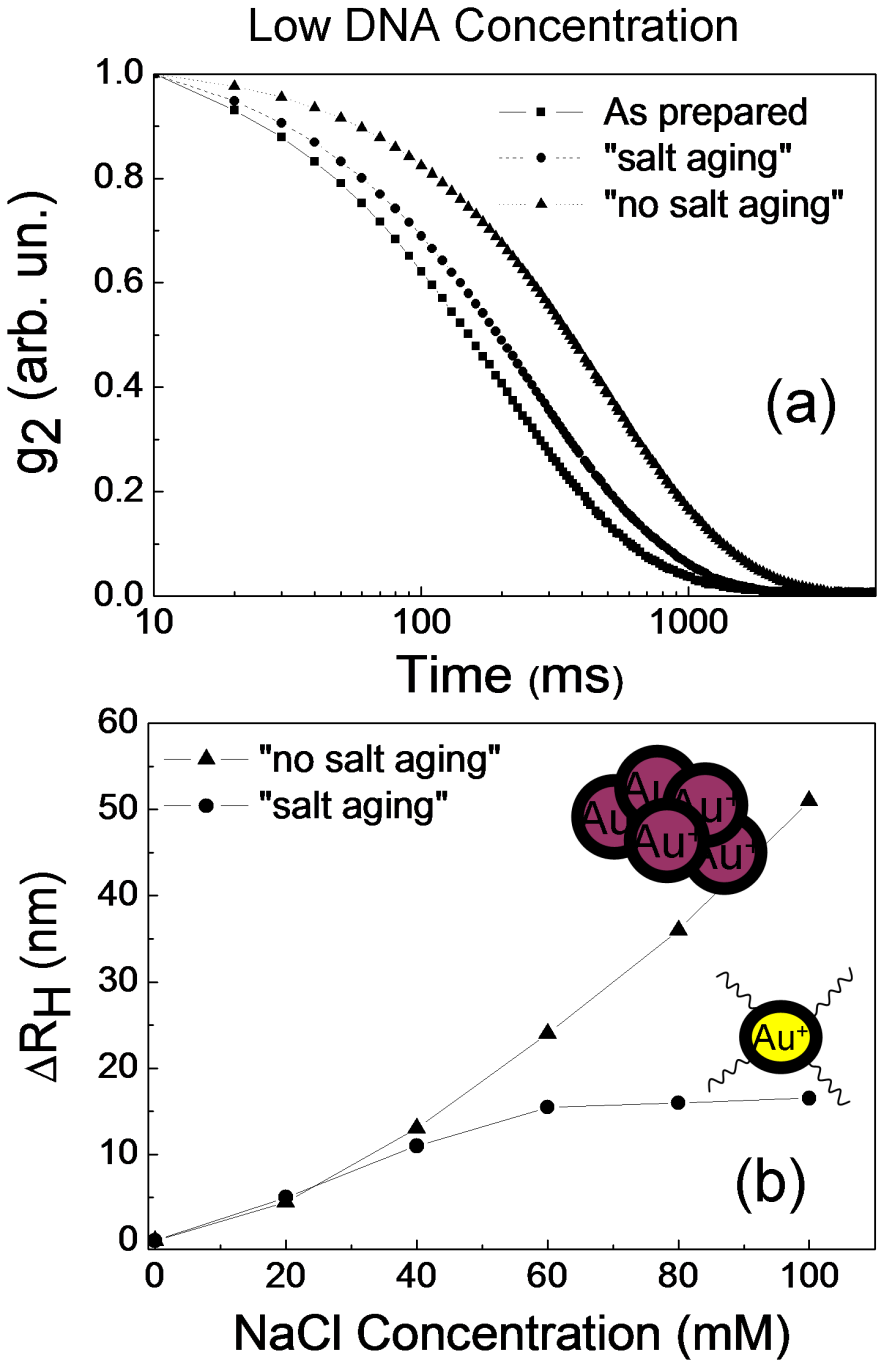
Autocorrelation functions g_2_ (t) of as-prepared AuNPs solution and of DNA/AuNPs solution at 100 mM NaCl, for “salt aging” and “no salt aging” procedure (a). Hydrodynamic radius difference (ΔR_H_) versus the NaCl concentration for “salt aging” and “no salt aging” procedure (b). The DNA concentration was 0.1 μM.

We have characterized the samples also by DLS measurements. In Fig. 3a we report the autocorrelation functions (g_2_) of as-prepared sample, as well as of DNA/AuNPs solutions at a salt concentration of 100 mM obtained with the “salt aging” and “no salt aging” procedures. In the DNA/AuNPs solutions we observe an increase of the decay time corresponding to an increase of the hydrodynamic radius of gold nanoparticle: the hydrodynamic radius is 20 nm in the as-prepared sample, becomes 38 nm and 72 nm in the DNA/AuNPs solutions for “salt aging” and “no salt aging” procedures, respectively. In order to better clarify the role played by the salt concentration and the aging process, we also carried out DLS measurements at different NaCl concentrations. The ΔR_H_ values versus the NaCl concentration are reported in Fig. 3b. The ΔR_H_ values for the sample obtained by the “no salt aging” procedure, increase continuously with NaCl concentration up to 52 nm. In the “salt aging” sample instead, it increases slowly (until 60 mM of NaCl) and then saturates at about 18 nm. This last result together with the UV-Vis spectra, reported in Fig. 2, evidences that in the “no salt aging” procedure the strong increase of ΔR_H_ is related to nanoparticles aggregation, while in the “salt aging” procedure the variation of ΔR_H_ is correlated to the DNA conjugation to the AuNPs. This radius variation is compatible with the length of our 50 Mer oligos (17 nm) used.

**Figure 4:**
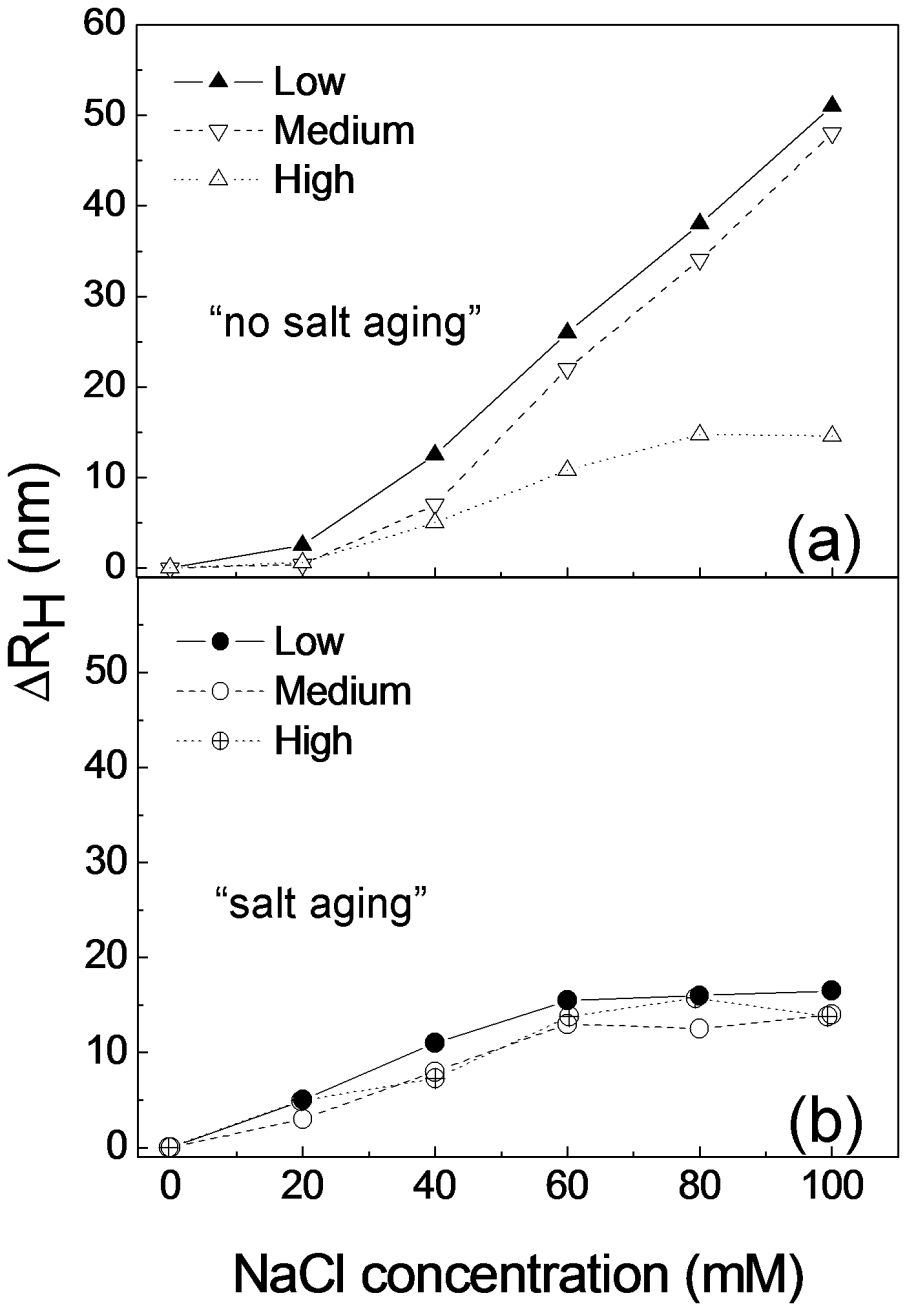
Hydrodynamic radius difference (ΔR_H_) versus the NaCl salt solution for different DNA concentrations, measured with “no salt aging” (a) and “salt aging” (b) procedure.

To shed light on the loading mechanisms, we also performed DLS measurements on DNA/AuNPs solutions at different DNA concentrations. The ΔR_H_ values versus NaCl concentration for the three different DNA concentrations used and for both “no salt aging” and”salt aging” procedures, are reported in Fig. 4.

For “no salt aging” (Fig. 4a) we observed an increase of the ΔR_H_ up to 52 nm for both “low” and “medium” DNA concentrations, whilst for the “high” DNA concentration we observe a saturation of ΔR_H_ at about 16 nm, suggesting that for the “low” and “medium” DNA concentration mainly AuNPS aggregation occurs, whereas for the “high” DNA concentration, the nanoparticles conjugation prevails.

In the case of “salt aging” procedure (Fig. 4b) the hydrodynamic radius difference increases when salt concentration goes up and saturates at a value of 18 nm, independently on the DNA concentration; this result indicates that the DNA/AuNPs conjugation is obtained for every DNA concentration. However the lowest DNA concentration (7.6 DNA strands for 1 nm^2^ of exposed gold surface) is enough to obtain a complete coverage of the nanoparticle surface.

This study reveals that DLS, by measuring the hydrodynamic properties of gold nanoparticles, allows, by determining the change in size, to validate this technique as powerful tool for directly evidencing their bio-conjugation.

The proposed technologies on such multimodal probes (e.g. DNA-AuNps) can provide an high impact in many fields giving molecular (e.g. biomarkers, gene expression) biological (e.g. metastasis, cell trafficking) and anatomical information.

Therefore, essential players in next generation biomedical techniques are nanoparticles-based multimodal imaging probes, which not only enhance imaging sensitivity and resolution but also possess specificity for so called “molecular imaging” capabilities. One of the advantages on nanoparticle probes is their flexibility when conjugated with DNA, peptides, and antibodies for monitoring specific molecular events such as gene expression and other biological processes as cell metastasis.

## Conclusions

The wide applicability of AuNPs in the biomaterial conjugation procedure, made crucial to synthesize high-quality and size controlled AuNPs, especially in DNA-based biodetection. In this study we assume to have reached this objective by conjugating DNA oligos to gold nanoparticles generated by laser ablation in water. We proved that DLS technique, coupled with the UV-Vis spectroscopy investigation, is a fast, reliable and convenient approach to monitor the DNA-gold nanoparticles conjugation through the measurements of their hydrodynamic properties.

Moreover laser generated nanoparticles are highly suitable for the conjugation process with low DNA concentration, either because of their “naked” surface due to the lack of capping species, or for their partial oxidation, that makes them more reactive and then more available for the conjugation processes in fact, for high enough concentration of DNA, conjugation occurs also with “non aging procedure”. More important, the reported results reveal that measurement of hydrodynamic properties of nanoparticles is a quite convenient tool to characterize bio-conjugation.

## References

[1] Garcia MA (2011) “Surface plasmons in metallic nanoparticles : fundamentals and applications” *J. Phys. D: Appl. Phys.* . 44: 283001–283042.

[2] Galletto P, Brevet PF, Girault HH, Antoine R, Broyer M (1999) “Enancement of the second harmonic response by asorbates on gold colloids: the effect of aggregation” *J. Phys.* . *Chem.B* 103: 8706–8710.

[3] Zhang YJ, Huang R, Zhu XF, Wang LZ, Wu CX (2012) “Synthesis, properties and optical applications of noble metal nanoparticle-biomolecule conjugates“ *Chinese Science Bullettin* . 57: 238–246.

[4] Blatchford CG, Campbell JR, Creighton JA (1982) “Plasma resonance-enhanced Raman cattering by adsorbates on gold colloids: the effects of aggregation” *Surf. Sci.* . 120: 435–455.

[5] Homola J, Sinclair SY, Gauglitz G (1999) “Surface plasmon resonance sensors: review” Sensor Actuat. B Chem. 54: 3–15.

[6] Sethi M, Knecht MR (2009) “Experimental studies on the interactions between Au nanoparticles and amino acids: bio-based formation of branched linear chains” *ACS Appl. Mater. Interf* . 1: 1270–1278. 10.1021/am900157m 20355923

[7] Wei F, Lam R, Cheng S, Lu S, Ho D, Li N (2010) “Rapid detection of melamine in whole milk mediated by unmodified gold nanoparticles” *Appl. Phys. Lett.* . 96: 133702–13704. 10.1063/1.3373325 PMC2859078 20428252

[8] Mirkin CA, Letsinger RL, Mucic RC, Storhoff JJ (1996) “A DNA based method for the rotational assembling nanoparticles into macroscopic materials”. *Nature* 382: 607–609. 8757129 10.1038/382607a0

[9] Ullien D, Cohen H, Porath D (2007) “The effect of the number of parallel DNA molecules on electric charge transport through standing DNA”. *Nanotechnology* 18: 424015–424019. 21730448 10.1088/0957-4484/18/42/424015

[10] Cuenca AG, Huabei MD, Jiang PD, Hochwald SN, Delano MD, et al (2006) “Emerging implications of nanotechnology on cancer diagnostics and therapeutic”. *Cancer* 107: 459–466. 16795065 10.1002/cncr.22035

[11] O’Neal DP, Hirsch LR, Halas NJ, Payne JD, West JL (2004) “Photo-thermal tumor ablation in mice using near infraredabsorbing nanoparticles” *Cancer Lett.* . 209: 171–176. 10.1016/j.canlet.2004.02.004 15159019

[12] Cai W, Gao T, Hong H, Sun J (2008) “Applications of gold nanoparticles in cancer nanotechnology” *Nanotech. Sci. and Appl* . 1: 17–32. 10.2147/NSA.S3788 PMC3808249 24198458

[13] Huang X, El-Sayed IH, Qian W, El-Sayed M (2006) “Cancer cell imaging and photothermal therapy in the near-infrared region by using gold nanorods” *J. Am. Chem.* . Soc (128) 2115–2120. 10.1021/ja057254a 16464114

[14] Le DV, Nguyen VT, Tang LJ, Jiang JH, Yu RQ, et al (2013) “Proteolysis-mediated protection of gold nanoparticles for sensitive activity assay of peptidases”. *Talanta* 107: 233–238. 23598217 10.1016/j.talanta.2013.01.016

[15] Lin J, Zhou Z, Li Z, Zhang C, Wang X, et al (2013) “Biomimetic one-pot synthesis of gold nanoclusters/nanoparticles for targeted tumor cellular dual-modality imaging” *Nanoscale Res. Lett* . 8: 170–174. 10.1186/1556-276X-8-170 PMC3637621 23587362

[16] Turkevich J, Stevenson PC, Hillier J (1951) “A study of the nucleation and growth processes in the synthesis of colloidal gold” *Discuss. Faraday. Soc.* . 11: 55–75.

[17] Majzik A, Patakfalvi R, Hornok V, De’ka’ny I (2009) “Growing and stability of gold nanoparticles and their functionalization by cysteine” *Gold Bull* . 42: 2–7.

[18] Martin MN, Basham JI, Chando P, Eah S (2010) “Charged gold nanoparticles in non-polar solvents: 10 min synthesis and 2D self-assembly”. *Langmuir* 26: 410–7417. 10.1021/la100591h 20392108

[19] Storhoff JJ, Elghanian R, Mucic RC, Mirkin CA, Letsinger RL (1998) “Colorimetric Differentiation of Polynucleotides with Single Base Imperfections Using Gold Nanoparticle Probes” *J. Am. Chem. Soc.* . 120: 1959–1964.

[20] Rongchao J, Guosheng W, Zhi L, Mirkin CA, Schatz GC (2003) “What Controls the Melting Properties of DNA-Linked Gold Nanoparticle Assemblies?” *J.Am.Chem.Soc* . 125: 1643–1654. 10.1021/ja021096v 12568626

[21] Hurst SJ, Lytton-Jean AKR, Mirkin CA (2006) “Maximizing DNA Loading on a Range of Gold Nanoparticle Sizes “ *Anal. Chem.* . 78: 8313–8318. 10.1021/ac0613582 PMC2525614 17165821

[22] Yang GW (2007) “Laser ablation in liquids: Applications in the synthesis of nanocrystals” *Prog. Mater. Sci* . 52: 648–698.

[23] Walter JG, Petersen S, Stahl F, Scheper T, Barcikowski S (2010) “Laser ablation-based one-step generation and bio-functionalization of gold nanoparticles conjugated with aptamers”. *J. Nanobiotech* 8: 251–258. 10.1186/1477-3155-8-21 PMC2939592 20731831

[24] Mafune’F, Kohno J, Takeda Y, Kondow T (2002) “Full physical preparation of size-selected gold nanoparticles in solution: laser ablation and laser-induced size control” J. *Phys. Chem.* . *B* 106: 7575–7579.

[25] Besner S, Kabashin AV, Winnik FM, Meunier M (2008) “Ultrafast laser based “green” synthesis of non-toxic nanoparticles in aqueous solutions” *Appl.Phys.* . *A* 93: 955–959.

[26] Sylvestre JP, Poulin S, Kabashin AV, Sacher E, Meunier M, et al (2004) “Surface Chemistry of Gold Nanoparticles Produced by Laser Ablation in Aqueous Media” *J. Phys. Chem.* . *B* 108: 16864–16869.

[27] Petersen S, Barcikowski S (2009) “Conjugation Efficiency of Laser-Based Bioconjugation of Gold Nanoparticles with Nucleic Acids” *J.Phys.Chem.* . *C* 113: 19830–19835.

[28] Haiss W, Nguyen TK, Thanh JA, Fernig DG (2007) “Determination of Size and Concentration of Gold Nanoparticles from UV-Vis” *Spectra Anal. Chem* . 79: 4215–4221. 10.1021/ac0702084 17458937

[29] Provencher SW (1982) “CONTIN: a general purpose constrained regularization program for inverting noisly linear algebraic and integral equation”. *Comput.Phys.Commun.* 27: 229–242.

[30] Berne BJ, Pecora R (1976) “Dynamic light scattering” (1976) Wiley, New York 376.

[31] Zimbone M, Calcagno L, Messina E, Baeri P, Compagnini G (2011) “Dynamic light scattering and UV–Vis spectroscopy of gold nanoparticles solution” *Mater. Lett.* . 65: 2906–2909.

[32] Zimbone M, Calcagno L, Baeri P, Messina G, Compagnini G (2012) ” Dynamic light scattering in gold colloids prepared by laser ablation in water” *Appl.Surf.Sci* . 258: 9246–9249.

[33] Shim JY, Gupta VK (2007) “Reversible aggregation of gold nanoparticles induced by pH dependent conformational transitions of a self-assembled polypeptide” *J. Colloid Interface Sci.* . 316: 977–983. 10.1016/j.jcis.2007.08.021 17825314

[34] Daizy P (2008) “Synthesis and spectroscopic characterization of gold nanoparticles”. *Spectrochim Acta Part A* 71: 80–85. 10.1016/j.saa.2007.11.012 18155956

[35] Mafune’ F (2004) “Structure diagram of gold nanoparticles in solution under irradiation of UV pulse laser” *Chem. Phys. Lett.* . 397: 133–137.

[36] Bohren CF, Huffman DR (1983), “Absorption and scattering of light by small particles” Wiley, New York.

[37] Kreibig U, Vollmer M (1908) “Optical properties of metal clusters” In: Toennies JP (ed) Springer series in Mater Sci, Springer, New York, (1995)

[38] Mie G (1908) ”Beiträge zur Optik trüber Medien, speziell kolloidaler Metallösungen” *Ann.* . *Phys* 25: 377–445.

[39] Sajti C, Petersen S, Manjón AM, Barcikowski S (2010) “In-situ bioconjugation in stationary media and in liquid flow by femtosecond laser ablation by femtosecond laser ablation” *Appl. Phys.* . *A* 101: 259–264.

[40] Jans H, Liu X, Austin L, Maes G, Huo Q (2009) “Dynamic Light Scattering as a Powerful Tool for Gold Nanoparticle Bioconjugation and Biomolecular Binding Studies” *Anal. Chem.* . 81: 9425–9432. 10.1021/ac901822w 19803497

[41] Hermanson GT (2008) “Bioconjugate Techniques” 2nd ed.; Published by Academic Press, Inc., pag.1202.

[42] Zhang X, Servos MR, Liu J (2012) ”Instantaneous and quantitative functionalization of gold nanoparticles with thiolated DNA using a pH-assisted and surfactant_free route”. *JACS* 134: 7266–7269. 10.1021/ja3014055 22506486

[43] Herdt AR, Drawz SM, Kang YJ, Taton TA (2006) “DNA dissociation and degradation at gold nanoparticle surfaces Colloids Surf. B”. 51: 130–139. 10.1016/j.colsurfb.2006.06.006 16879950

[44] Bhatt N, Huang PJ, Dave N, Liu J (2011) “Dissociation and Degradation of Thiol-Modified DNA on Gold Nanoparticles in Aqueous and Organic Solvents”. *Langmuir* 27: 6132–6137. 21513322 10.1021/la200241d

[45] Liu J, Lu Y (2006) ”Preparation of aptamer-linked gold nanoparticle purple aggregates for colorimetric sensing of analytes” *Nat. Protoc* . 1: 246–252. 10.1038/nprot.2006.38 17406240

[46] Hill HD, Millstone JE, Banholzer MJ, Mirkin CA (2009) “The Role Radius of Curvature Plays in Thiolated Oligonucleotide Loading on Gold Nanoparticles,”. *ACS Nano* 3: 418–424. 19236080 10.1021/nn800726e PMC3241534

[47] Zu Y, Gao Z (2009) “Facile and Controllable Loading of Single-Stranded DNA on Gold Nanoparticles” *Anal.Chem.* . 81: 8523–8528. 10.1021/ac901459v 19751052

